# Mental Health Practitioners’ Understanding of Speech Pathology in a Regional Australian Community

**DOI:** 10.3390/healthcare9111485

**Published:** 2021-11-01

**Authors:** Tina Janes, Tania Signal, Barbra Zupan

**Affiliations:** School of Health, Medical and Applied Sciences, Central Queensland University, Rockhampton, QLD 4701, Australia; t.signal@cqu.edu.au (T.S.); b.zupan@cqu.edu.au (B.Z.)

**Keywords:** communication, mental health, awareness of speech pathology

## Abstract

(1) Background: This study aimed to determine the level of knowledge and the perceptions of speech pathology held by a sample of regional mental health practitioners and to explore factors that facilitate understanding of the roles of speech pathologists in mental health. While mental health is recognised as an area of practice by Speech Pathology Australia, the inclusion of speech pathologists in mental health teams is limited. (2) Methods: An anonymous online survey was created using previously validated surveys and author generated questions and distributed to mental health practitioners in Central Queensland, Australia. (3) Results: Mental health practitioners had difficulty identifying speech pathology involvement when presented with case scenarios. Accuracy was poor for language-based cases, ranging from 28.81% to 37.29%. Participants who reported having worked with a speech pathologist were more likely to demonstrate higher scores on the areas of practice questions, [*r*(53) = 0.301, *p* = 0.028], and the language scenarios [*r*(58) = 0.506, *p* < 0.001]. They were also more likely to agree to statements regarding the connection between speech pathology and mental health, *r*(59) = 0.527, *p* < 0.001. (4) Conclusions: As found in this study, contact with speech pathologists is a strong predictor of mental health providers’ knowledge of the speech pathology profession. Thus, the challenge may be to increase this contact with mental health providers to promote inclusion of speech pathologists in the mental health domain.

## 1. Introduction

The World Health Organisation (WHO) defines mental health as “a state of well-being in which an individual realises their own abilities, can cope with the normal stresses of life, work productively and make a contribution to their community” [1]. In Australia, 17.5% of adults reported having a mental or behavioural condition, with women reporting at 19.2% and men at 15.8% [2]. Anxiety disorders were most commonly reported (11.2%), followed by mood (affective) disorders, including depression (9.3%) [2]. Mental health, and the promotion of mental health, is an integral part of public health and thus a concern for consumers, communities, professionals, and governments [1]. Law et al. [3] emphasised the importance of speech pathologists (SPs) being included in the public health discourse on the management of mental health, as communicative competence is central to successful relationships, social engagement and employment skills, all key determinants of mental health. Speech Pathology Australia state that “assessment, diagnosis and treatment of communication and swallowing difficulties of individuals with, or at risk of, mental illness is essential and within the scope of practice of speech pathologists” [4]. The promotion of and justification for SPs’ inclusion in interprofessional mental health teams requires an understanding of the complex and multifactorial relationship between communication, swallowing, and mental health disorders and the roles of SPs in their assessment and management [4].

Research has identified a link between developmental language disorders in childhood and poor mental health and well-being outcomes in adulthood [3,5,6,7,8,9]. Developmental language disorders continue to affect participation in cognitive and mental health assessments and management because communication (verbal and non-verbal) skills are integral to data gathering and treatment for mental health disorders, as well as establishing the therapeutic relationship necessary for positive health outcomes [10]. The diagnostic overlap between communication and psychological disorders validates the involvement of SPs in both the assessment and management of mental health disorders [11]. For example, communication and/or swallowing deficits are associated with disorders such as schizophrenia, feeding, and eating disorders and neurocognitive disorders (i.e., dementias) [12,13]. Interprofessional collaboration between SPs and traditional mental health clinicians, such as psychologists, has the potential to assist differential diagnosis of communication and swallowing difficulties embedded in the mental health profile and facilitate a communication friendly therapeutic environment [11].

SPs are routinely involved in the management of cognitive, communication, swallowing, and speech disorders that exist comorbidly with mental health disorders. This includes diagnoses such as traumatic brain injury, Parkinson’s disease, stroke, autism spectrum disorder, Fragile X syndrome, and stuttering, which all are commonly associated with mental health disorders, particularly depression and anxiety [13,14,15,16]. It is apparent that many disorders within the typical areas of practice for SPs present with increased risks for mental health disorders and associated adverse psychosocial outcomes, including unemployment and incarceration. This reinforces the need to further investigate the routine inclusion of speech pathologists in interprofessional mental health teams, because anecdotal evidence suggests that this is currently not occurring, particularly in rural and regional areas.

The National Disability Insurance Scheme (NDIS) provides funding to an estimated 500,000 Australians who have permanent and significant disability, including the psychosocial supports funding for individuals with mental health conditions [17]. The progressive rollout of the NDIS across Australia accentuates the need for mental health providers and participants in the NDIS to be cognisant of available services and to appropriately access these services. The inclusion of an interprofessional approach to the management of mental health disorders in children, adolescents, and adults is discussed in the literature, but with notable absence of specific reference to the speech pathology profession [18,19,20,21]. In these studies, allied health professionals such as occupational therapists and psychologists were explicitly listed as team members, but SPs were not. The absence of SPs supports anecdotal evidence that SPs are not typically included in, or considered as core members of, interprofessional mental health teams on a global level.

In the Australian context, The Better Access to Mental Health Care initiative was introduced to Medicare in November 2006 to improve outcomes for people with common mental health disorders by offering a multidisciplinary approach [22]. Medicare rebates were made available for psychiatrists, psychologists, social workers, and occupational therapists, but not SPs [22]. SPs were also not listed as a service to support Australian children and adolescents’ mental health and wellbeing in the 2015 Mental Health report [23]. This lack of inclusion as core mental health providers reiterates the need for increased consumer and professional awareness of the role that SPs can have in the holistic assessment and management of communication and swallowing disorders within the mental health context. It is possible they have not been included thus far due to a poor understanding of SPs’ areas of practice, particularly when these practice areas are presented within a real-life scenario (i.e., the vignettes). In a survey of community members, Janes et al., [24] found that disorders of language were poorly recognised as falling within the areas of practice for SPs, with only 20% of participants identifying language delays as a reason to see a SP. Similar to previous research (Janes et al. [25]; Mahmoud et al. [26]) also noted that while participants initially indicated a knowledge of SPs areas of practice (via yes/no questioning), actual knowledge (tested via vignettes) was lacking. If the scope of practice for speech pathology is not well understood in the community, it is possible that mental health providers may also not fully understand the various services SPs offer, which will impact appropriate referrals to, and access of, relevant services.

The aims of this study are to determine mental health practitioners’ level of knowledge, their perceptions of SPs, and to explore factors that facilitate understanding of SPs’ areas of practice and role in mental health. For this study mental health practitioners were identified as those included in the Medicare’s Better Access to Mental Health Care [22] document and acknowledged by organisations such as Psychotherapy and Counselling Federation of Australia [27] and Allied Health Professions Australia [28]. The study focused on a sample of mental health practitioners in Central Queensland, which includes regional, rural, remote, and very remote demographics. This region was specifically targeted because these demographics are associated with reduced access to health care services and increased risk of poor mental health [29]. Early access to services at the onset of a mental illness is crucial and may even prevent some mental illnesses from occurring [30].

This study proposes the following three research questions:

1. How knowledgeable are mental health practitioners of speech pathologists’ broad areas of practice? It is hypothesised that mental health practitioners will be less able to identify areas of speech pathology practice when a condition is embedded in a scenario versus a yes/no format. We also hypothesised that they will have greater knowledge of speech related disorders (e.g., speech delay, voice, and stuttering) compared to developmental language disorders.
2. How aware are mental health practitioners of the specific involvement of speech pathologists in the mental health arena? It is hypothesised that mental health practitioners will have limited awareness of the impact of developmental language disorders on mental health, the co-existence of communication disorders with mental health disorders and the increased risk of swallowing disorder in people with mental health conditions.
3. What factors facilitate knowledge of speech pathologists’ areas of practice and role in mental health? It is hypothesised that a range of employment factors (e.g., years of experience, employment settings) will facilitate greater knowledge of SPs’ areas of practice and role in mental health.

## 2. Materials and Methods

### 2.1. Survey Development

The survey included 27 questions organised into four sections (Supplementary Material). The first section gathered information about the respondent and their personal experiences with SPs. The remaining three sections collated information on participants’ knowledge of speech pathology. Firstly, participants used a one-to-five Likert scale to rate the likelihood of SP involvement for 18 general areas of practice such as attention and concentration, play and imaginative skills, social communication, theory of mind, and critical thinking skills. SP involvement was appropriate for all 18 areas. The next section included seven vignettes, derived and used in previous studies on public awareness of speech pathology [25,26,31,32]. Each vignette described a child with a speech or language delay and participants had to indicate whether the child would require SPs’ intervention. The final section included seven author generated statements relating to the involvement of SPs in mental health (e.g., “There are higher prevalence rates of swallowing disorders in people with diagnosed mental health conditions”) and participants needed to rate how strongly they agreed with the statement.

### 2.2. Survey Dissemination

Ethical approval for the current study was granted by the appropriate ethics committee, project number H17/05-073. Data was collected online using the SurveyMonkey platform. The survey link was sent by email to mental health facilities, and other psychologists and occupational therapists in the Central Queensland region. Recipients were encouraged to forward the link to other professionals working within mental health.

### 2.3. Data Analysis

Quantitative data were analysed using the IBM Statistical Package for the Social Sciences Statistics (version 26, IBM, NY, USA). Due to the aims of the study, responses from SPs were excluded. The professions of social work and counsellors were recoded as ‘counselling’; and pharmacy, nursing, and medical were recoded as ‘medical’. In addition to individual scores for the vignettes, a combined score was calculated for responses to speech versus language vignettes. An overall accuracy score was also calculated for areas of practice questions and SPs in mental health statements, with higher scores corresponding to greater accuracy. The analyses employed in this study comprised descriptive measures and parametric tests including Pearson’s correlation (*r*), an independent *t*-test (*t*), and one-way analysis of variance (ANOVA).

## 3. Results

### 3.1. Participants

Sixty-one mental health professionals from Central Queensland, Australia completed the online survey. Table 1 outlines professional details of the participants. As shown, approximately two-thirds of participants were psychologists or occupational therapists (64%, *n* = 39). A significant majority of participants did not have a speech pathologist on their team, (68.85%, *n* = 42), *t*(60) = 5.210, *p* < 0.001. Despite this, 70.49% (*n* = 43) reported that they knew what speech pathologists do (self-reported knowledge); 60.65% (*n* = 37) said they knew when to refer to SPs; and 72.1% (*n* = 44) agreed that having a speech pathologist as a member of a mental health team would be valuable.

### 3.2. Research Question 1—How Knowledgeable Are Mental Health Practitioners of SPs’ Broad Areas of Practice?

As evident in Table 2, the areas of practice that were most frequently identified as necessitating speech pathology input were difficulties with speech, voice, oral motor/swallowing, receptive, and expressive language. Conversely, the areas least identified were impulse control, extraneous bodily movements, attention and concentration, medication profile, and comorbidities. The results from the paediatric vignettes (Table 3) indicated that overall, accurate identification of SPs’ involvement in various clinical cases was low, ranging from 25.42% for a scenario describing a voice disorder to 54.24% for one describing an articulation disorder (i.e., lisp). Both scenarios would be classified as speech disorders. Identification of the need for SPs involvement in cases describing language (including literacy) disorders was also low, ranging from 28.81% to 37.29% (Table 3).

### 3.3. Research Question 2—How Aware Are Mental Health Practitioners of the Specific Involvement of SPs in the Mental Health Arena?

Participants were asked to rate their level of agreement for seven statements related to SPs and mental health (Table 4). Most participants accurately responded to five of the seven statements. These statements were related to (1) the connection between behavioural disorders, psychological trauma, and children in care to communication difficulties; (2) the increased likelihood of mental health concerns for those with childhood language difficulties; and (3) the need for more speech pathologists’ to be involved in mental health care programs. Participants disagreed more than agreed to the two statements regarding the link between speech pathology and mental health (see statements 4 and 7 in Table 4).

### 3.4. Research Question 3—What Factors Influence Knowledge of SPs’ Areas of Practice and Roles in Mental Health?

Normality of data was confirmed via visual inspection of quantile-quantile (QQ) plots. To explore whether participants’ accuracy on the scope of practice questions was related to their knowledge, experience, and attitudes to speech pathology, Pearson correlation coefficients were conducted. Results showed participants’ self-reported knowledge, *r*(53) = 0.391, *p* = 0.004; confidence in referring to a speech pathologist, *r*(53) = 0.494, *p* < 0.001; perceived value of a SPs, *r*(53) = 0.328, *p* = 0.017 and having worked with a speech pathologist before, *r*(53) = 0.301, *p* = 0.028 were significantly related. No significant relationships were found with any of the remaining variables (i.e., profession, experience, workplace, having a speech pathologist on the team). In addition, there was no significant effect of any of the independent variables on accuracy for the speech vignettes as a whole. The factors that were significantly related to participants’ greater accuracy on the language vignettes were having a speech pathologist on their team, *r*(58) = 0.331, *p* = 0.011; self-reported knowledge of speech pathology, *r*(58) = 0.531, *p* < 0.001; confidence in referring to a speech pathologist, *r*(58) = 0.486, *p* < 0.001; perceived value of a speech pathologist to the mental health team, *r*(58) = 0.434, *p* = 0.001; and having worked with a speech pathologist before, *r*(58) = 0.506, *p* < 0.001. Additionally, profession also impacted, with occupational therapists performing significantly better than the other professions on the language vignettes, *F*(3,54) = 5.182, *p* = 0.003.

Participants’ accuracy on mental health statements was significantly related to having a speech pathologist on their team, *r*(59) = 0.331, *p* = 0.010; self-reported knowledge of SPs, *r*(59) = 0.625, *p* < 0.001; confidence in referring to a speech pathologist, *r*(59) = 0.372, *p* = 0.001; perceived value of SPs, *r*(59) = 0.689, *p* < 0.001 and having worked with a speech pathologist before, *r*(59) = 0.527, *p* < 0.001. Additionally, one-way ANOVAs were conducted to note significant differences between the means of the relevant variables. The results indicated that participants who had worked with SPs before were significantly more confident in referring to a speech pathologist, *F*(4,56) = 20.075, *p <* 0.001 and they had a significantly greater self-reported knowledge of speech pathology, *F*(4,56) = 20.276, *p <* 0.001. They also placed significantly greater value on the role of SPs in mental health, *F*(4,56) = 9.133, *p <* 0.001 compared to mental health practitioners who had not worked with SPs.

## 4. Discussion

The aim of this study was to determine the level of knowledge and the perceptions of SPs held by a sample of mental health practitioners in Central Queensland. Speech Pathology Australia [1,12] endorses the inclusion of SPs in mental health teams, with SPs playing a critical role in the assessment and management of communication and swallowing disorders for both children and adults within the mental health contexts. Despite this endorsement, the current study shows that SPs do not appear to be routinely included in mental health teams with over two-thirds of the sample reporting that they do not currently work directly with SPs.

Overall, it appears as though the mental health practitioners who completed this survey know that speech, language, and swallowing are core business for SPs, as indicated by their high level of accuracy in identifying these areas as falling within SPs’ scope of practice. However, almost 52% (*n* = 30) of participants were unsure or disagreed that SPs worked with attention and concentration, and almost 64% (*n* = 37) either disagreed or were unsure that impulse control was within SPs’ areas of practice. This is at odds to the fact that SPs regularly work with attention, concentration, and impulse control difficulties as part of the profiles associated with autism spectrum disorders, attention deficit hyperactivity disorder, traumatic brain injury, and eating disorders [1].

The two areas least recognised by participants as within the areas of practice for SPs were extraneous bodily movements and medication profile. Extraneous bodily movements are present in many conditions SPs work with and often function as key markers for differential diagnosis of the dysarthrias (motor speech disorders) and types of neurological conditions [33]. However, 79% (*n* = 46) of participants did not acknowledge this as an area to be considered by SPs. In addition, 77.58% (*n* = 45) of participants did not think speech pathologists should be concerned with a patient’s medication profile. Medication induced dysphagia and other movement disorders either caused or were exacerbated by antipsychotic medications put patients at a much greater risk of swallowing disorders, a potentially life limiting condition that requires the expertise of SPs.

As hypothesised, mental health practitioners showed less knowledge of SPs’ areas of practice when the condition was embedded in a scenario (i.e., vignettes), identifying the need for speech pathology input less than half the time for all vignette cases except the one focused on a child with a lisp. However, the hypothesis that mental health practitioners would have a greater knowledge of speech related disorders compared to developmental language disorders was not supported as mental health practitioners showed similar difficulties for both types of vignettes. This reduced knowledge of SP practices is an area that needs further research as communication disorders (particularly language) are known to have a negative and lifelong impact on an individual’s educational and occupational achievement and psychosocial well-being [4,5].

Participants successfully identified that psychological trauma can negatively impact language development and that children in care are at a much greater risk of having a language disorder, social and emotional difficulties, and increased risk of contact with the criminal justice system. However, when participants were asked to rate their agreement to the statement that there is an increased likelihood of mental health concerns in those who initially presented with significant speech/language impairments as a child, they performed poorly. As noted above and in the introduction, there is a growing body of evidence supporting the link between a developmental language disorder as a child and mental health disorders as an adult [3,5,6,7,8,9]. These results suggest that mental health practitioners are aware of the association between language disorders in children who have experienced trauma, including being in care, but that this did not translate to an awareness of the long-term impact of developmental language disorders on mental health and well-being.

Only 25% (*n* = 15) of participants strongly agreed that there are many DSM-5 categories which include communication impairment in the diagnostic criteria even though the latest edition of the DSM-5 and ICD-11 classifications include a large number of disorders (e.g., depressive and anxiety disorders; trauma and stressor related disorders; neurocognitive disorders) as having associated communication and/or swallowing deficits and therefore within the realm of practice for SPs 12,34 Interprofessional collaboration between SPs and traditional mental health clinicians will assist differential diagnosis of communication and swallowing difficulties and enable appropriate treatment strategies.

Our third research question focused on factors that may facilitate or impede knowledge of SPs’ areas of practice and role in mental health. We had hypothesised that a range of employment factors might influence responses, including profession. However, profession was only found to significantly influence responses to the language vignettes with occupational therapists found to be more knowledgeable of paediatric developmental language disorders than other professionals in the study. This implies that profession is largely not a predictor of awareness of communication and swallowing disorders and that education efforts need to target all mental health practitioners equally. Additionally, neither setting nor length of experience as a mental health clinician affected knowledge of the role of SPs, thus all workplaces/experience levels need to be targeted in awareness campaigns.

The two most influential factors in knowledge of the areas of practice for SPs included having a speech pathologist on the team and having worked with a speech pathologist previously. When either of these two factors were present, mental health practitioners were found to be more accurate on the language vignettes and the speech pathology in mental health statements. Moreover, those who had previously worked with SPs were also more accurate on the areas of practice questions. The presence of these two factors also resulted in greater confidence in referring to SPs; assigning a higher value on SPs’ services in the mental health context and a superior self-reported knowledge of speech pathology. Mental health practitioners who have worked with SPs could be good advocates for the role of SPs in this context, but advocacy needs to extend to policy makers at national and international levels as the exclusion of SPs in the mental health arena is not uniquely Australian [18,21]. Perceiving speech and language therapy services as part of the public health umbrella, and hence promoting a health and well-being service delivery model for SPs, may be one way to facilitate inclusion on mental health teams [4].

One avenue in which recognition of the role that SPs have in the holistic assessment and management of people with mental health conditions is through the NDIS psychosocial supports funding. The psychosocial supports umbrella stipulates six core aspects of functional capacity, all of which are related to SPs. Despite SPs being excluded from previous funding for mental health supports, the NDIS psychosocial support funding opens the door for SPs to become integral members of mental health teams. However, for appropriate inclusion of SPs to occur, suitable referrals and requests for service must be made by the participant and/or service providers. This, in turn, requires enhanced appreciation of the roles of SPs, particularly with respect to communication, social interaction, learning, and self-management.

## 5. Conclusions

There is a growing body of evidence that supports a diagnostic overlap between communication and mental health disorders, psychosocial comorbidities, and the increased risk of dysphagia in individuals with mental health disorders. Despite this, SPs are not routinely included in mental health teams in Australia. This study aimed to contribute to the limited research on the inclusion of SPs in mental health teams and it highlighted the need for further research into SPs’ role in adolescents and adults with mental health conditions. The mental health practitioners surveyed in this study were most knowledgeable when SPs’ areas of practice (i.e., speech, oral motor, voice, and developmental language disorders) were explicitly identified. Knowledge of these same disorders was considerably lower when they were embedded within a scenario. This translates to a real-life context where mental health practitioners are presented with a case and need to make appropriate judgments about who should comprise the interprofessional mental health team. If mental health practitioners cannot recognise the signs of speech and language disorders, they are unlikely to include SPs on the team. Thus, a lack of exposure to SP compounds results in minimal inclusion of SPs in mental health teams. The challenge for the profession of speech pathology is to increase contact with mental health facilities and practitioners so that SPs can advocate for their routine inclusion in the assessment and management of people with mental health disorders.

## Figures and Tables

**Table 1:** Participants.

Participant Details	*n*	%
Profession		
Psychologist	22	36.1
Occupational therapist	17	27.9
Social worker/counsellor	12	19.7
Other (nursing, pharmacy, medical)	10	16.4
Experience		
Less than 5 years	14	23
5 to 10 years	18	29.5
11 to 15 years	14	23
16 to 20 years	7	11.5
More than 20 years	8	13.1
Workplace		
Multiple workplaces	15	24.6
Mental health facility only	14	23.0
Private practice only	8	13.1
Community health only	7	11.5
Non-government organisation only	7	11.5
Other only (e.g., university, schools)	7	11.5
Hospital only	3	4.9
Age Groups Worked with		
Under 5 years	24	39.34
5 to 12 years	34	55.74
13 to 16 years	39	63.93
17 to 19 years	50	81.79
20 to 30 years	36	59.02
31 to 40 years	34	55.75
41 to 50 years	32	52.46
Over 50 years	59	96.72
Nature of Role		
Intervention	52	85.25
Assessment/diagnosis	47	77.05
Family supports	35	57.38
Case management	32	52.46
Community supports	27	44.26
Student supervision	25	40.98
Educational supports	21	34.43
Team management	17	27.87
Emergency/frontline support	16	26.23
Vocational supports	15	24.59
Other	3	4.92
Team members		
Psychologists	49	80.3
Social workers and counsellors	46	75.41
Nursing staff	36	59
Occupational therapists	36	59
Psychiatrists	30	49.2
Indigenous health workers	29	47.54
Other (e.g., community workers, SLPs)	14	23
Teachers	13	21.3
Allied health assistants	11	18
General practitioners	11	18
Guidance officers	7	11.5

**Table 2:** Areas of practice for speech pathologists.

Area of Practice	Strongly Agree		Agree		Unsure		Disagree		Strongly Disagree	
	%	*n*	%	*n*	%	*n*	%	*n*	%	*n*
Attention and concentration	5.17	3	43.1	25	32.76	19	13.79	8	5.17	3
Impulse control	0	0	36.21	21	36.21	21	22.41	13	5.17	3
Emotional literacy	36.21	21	44.83	26	12.07	7	5.17	3	1.72	1
Play skills	19.3	11	40.35	23	26.32	15	12.28	7	1.75	1
Pragmatics	48.28	28	39.66	23	6.90	4	1.72	1	3.45	2
Parental attachment	22.41	13	55.17	32	18.97	11	3.45	2	0	0
Extraneous bodily movements	3.45	2	17.24	10	48.28	28	24.14	14	6.90	4
Dysarthria	47.37	27	17.54	10	29.82	17	1.75	1	3.51	2
Oral motor/dysphagia	70.69	41	25.86	15	3.45	2	0	0	0	0
Co-morbidities	19.3	11	36.84	21	40.35	23	3.51	2	0	0
Medication profile	5.17	3	17.24	10	50	29	22.41	13	5.17	3
Receptive language	67.24	39	27.59	16	3.45	2	0	0	1.72	1
Expressive language	71.19	42	25.42	15	1.69	1	0	0	1.69	1
Social cognition	27.59	16	41.38	24	25.86	15	3.45	2	1.72	1
Literate language	65.52	38	27.59	16	3.45	2	0	0	3.45	2
Critical thinking	39.66	23	36.21	21	17.24	10	3.45	2	3.45	2
Speech	78.95	45	17.54	10	1.75	1	0	0	1.75	1
Voice	74.14	43	20.69	12	3.45	2	0	0	1.72	1

**Table 3:** Paediatric vignette scores.

Disorder	Yes		Probably		Unsure		Probably No		No	
	%	*n*	%	*n*	%	*n*	%	*n*	%	*n*
Speech cluster										
Articulation disorder (5-year-old)	54.24	32	20.34	12	10.17	6	15.25	9	0	0
Stuttering (3-year-old)	32.2	19	38.98	23	16.95	10	8.47	5	3.39	2
Voice disorder (8-year-old)	25.42	15	28.81	17	32.2	19	6.78	4	6.78	4
Language cluster										
Language delay (15 months)	37.29	22	15.25	9	27.12	16	18.64	11	1.69	1
Language delay (3-year-old)	29.31	17	15.52	9	13.79	8	27.59	16	13.79	8
Language delay (7-year-old)	35.59	21	30.51	18	13.56	8	15.25	9	5.08	3
Literacy difficulties (8-year-old)	28.81	17	28.81	17	18.64	11	22.03	13	1.69	1

**Table 4:** Beliefs regarding communication and swallowing disorders.

Statement	Strongly Agree		Agree		Neutral/Unsure		Disagree		Strongly Disagree	
	%	*n*	%	*n*	%	*n*	%	*n*	%	*n*
1. There is an increased likelihood of mental health concerns in those who initially presented with significant speech/language impairments as a child	27.12	16	42.37	25	25.42	15	5.08	3	0	0
2. Behavioural disorders may indicate undiagnosed communication, learning, literacy, and/or attention/concentration problems	59.32	35	33.9	20	6.78	4	0	0	0	0
3. Psychological trauma can negatively impact language development	62.71	37	33.9	20	3.39	2	0	0	0	0
4. There are higher prevalence rates of swallowing disorders in people with diagnosed mental health conditions	11.86	7	13.56	8	67.8	40	6.78	4	0	0
5. There are many DSM-5 diagnostic categories which include communication impairment in the diagnostic criteria	25.42	15	45.76	27	28.81	17	0	0	0	0
6. Children in care are at a much greater risk of having a language impairment, social, and emotional difficulties and increased risk of contact with the criminal justice system	62.71	37	28.81	17	8.47	5	0	0	0	0
7. There is a greater need for SLPs to be involved in mental health care programs for children and adults	52.54	31	23.73	14	22.03	13	1.69	1	0	0

## Data Availability

Not applicable.

## Supplementary Materials

The following are available online at https://www.mdpi.com/article/10.3390/healthcare9111485/s1, File S1: Mental health practitioners’ awareness of speech pathology survey.
