# Supplementary material for: Mental Health Practitioners’ Understanding of Speech Pathology in a Regional Australian Community
Source: Healthcare (Basel). 2021 Nov 1;9(11):1485. doi: 10.3390/healthcare9111485 (PMC8622772; doi:10.3390/healthcare9111485)
Supplement: Supplementary file 1 [file healthcare-09-01485-s001.zip › healthcare-1407321-supplementary-update.pdf]

File S1. Mental health practitioners' awareness of speech pathology survey.

Question 1. I am, or have previously been involved in the provision of mental health services.

- Yes
- No

Question 2. What is your profession?

- Psychologist
- Occupational therapist
- Registered nurse
- Social Worker
- Psychiatrist
- Pharmacist
- Counsellor
- Dietitian
- Other (please specify)

Question 3. How long have you been in this position / profession?

- Less than five years
- Five to 10 years
- 11 to 15 years
- 16 to 20 years
- 21 to 25 years
- More than 25 years

Question 4. Select your current workplace/s (more than one can be selected).

- Private practice
- Mental health facility
- Hospital – acute services
- Hospital – rehabilitation
- Community health
- Non-Government organisation
- School
- University
- Disability services
- Correctional services
- Depart of community services
- Other (please specify)

Question 5. Please select the age groups of clients you provide a service to.

- Under five years
- Five to 12 years
- 13 to 16 years
- 17 to 19 years
- 20 to 30 years
- 31 to 40 years
- 41 to 50 years
- 51 to 65 years

- Over 65 years

Question 6. Please select as many of these that describe the nature of your role.

- Assessment / Diagnosis of Mental Health concerns
- Intervention for mental health concerns
- Community Supports
- Family supports
- Vocational supports
- Educational supports
- Case management
- Team management
- Student supervision
- Emergency / Frontline support

Question 7. Please select any of the professions below that are a part of your team.

- Psychologists
- Social Workers
- Indigenous liaison officers
- Indigenous health workers
- Allied health assistants
- Registered nurses
- Psychiatrists
- Occupational therapists
- General Practitioners

- Teachers
- Guidance Officers
- Counsellors
- Other (please specify)

Question 8. Do you have a speech pathologist as a member of your team?

- Yes
- No

Question 9. Is this speech pathologist based on site or a different location?

- On site
- A different location
- A combination

Question 10. Select as many of the below that apply to how you work with the speech pathologist.

- Referral for assessments
- Referral for intervention
- As a part of the team
- As an outreach service
- Family consultancy
- School consultancy
- Other (please describe other ways)

Question 11. You answered 'no' to having a speech pathologist on your team, but do you have a speech pathologist that you would refer to if you thought the need arose?

- Yes
- There are a few I would refer to depending on the situation
- Not really
- No

Question 12. Do you feel confident in knowing when to refer to a speech pathologist?

- Always
- Mostly
- Sometimes
- Rarely
- Not at all

Question 13. Please indicate the extent to which you agree with this statement - "Having a speech pathologist perform speech pathology roles in a mental health team would be useful".

- Strongly agree
- Agree
- Somewhat agree / unsure
- Disagree
- Strongly disagree

Question 14. Select the one answer that best applies to this statement - "I know what speech pathologists do".

- Strongly agree
- Agree
- Somewhat agree / unsure
- Disagree
- Strongly disagree

Question 15. I have worked with speech pathologists before.

- Yes – a lot
- Yes – some
- Yes – a little
- Rarely
- No

Question 16. Please list the settings in which you have worked with speech pathologists (select as many that apply).

- Education - Primary or Secondary
- Private Practice
- Community Health
- Hospital
- University / Teaching facilities
- Disability Services

- Mental Health Services
- Non-Government Agencies
- Other (please specify)

Question 17. Please select the answer that best describes your experience when working with a speech pathologist.

- Very positive
- Positive
- Somewhat positive
- Not very positive
- Unsure
- Other

Question 18. Other types of contact I have had with speech pathologists include: (please select all that apply).

- Personal experience
- Conferences / workshops
- Interagency meetings
- Educational settings
- Other (please specify)

Question 19. Rate the below areas as they apply to this statement - "A speech pathologist is involved in the assessment and intervention of...........".

- Attention and concentration
- Impulse control
- Emotional literacy/use of language to support thoughts and feelings
- Play and imaginative skills
- Pragmatic awareness and use (verbal and non-verbal)
- Social communication and interaction skills
- Parental/caregiver dyad/attachment, interactions both verbally and non-verbally
- Extraneous bodily movements
- Dysarthria
- Oral motor skills, dysphagia (swallowing difficulties) and/or impaired saliva control
- Co-morbidities
- Medication profile
- Receptive language
- Expressive language
- Theory of mind development and social cognition skills
- Language/phonological awareness/literacy abilities
- Critical thinking skills/verbal problem solving abilities
- Speech (phonology/articulation)

- Voice: volume, fluency, prosody, tone

Question 20. Please indicate whether you think the children in the following vignettes should be seen by a speech pathologist.

- Vignette 1: Shannon, age 5, can't say the 's' sound properly, and uses the 'th' sound instead. For example she says 'thand' for 'sand'.
- Vignette 2: Raymond, age 8, has a lot of trouble with reading and writing, but he speaks well with his friends and family. The school plans to give him a group of tests to find the cause/s of the problem.
- Vignette 3: Kylie, age 3, uses about 50 different words and is starting to put words together like 'want cookie'. She understands simple questions and can point to pictures and parts of her body when you name them.
- Vignette 4: Jason, age 8, has a husky voice. His mother says he's always yelling.
- Vignette 5: Sally, who is 15 months old, doesn't understand simple phrases like 'come here' or 'don't touch'.
- Vignette 6: Wayne, age 3, sometimes repeats sounds or words when he's excited but he never seems to notice it. For example, he might say, 'l-l-l-look at me, Mummy!'. Wayne's parents are not concerned about his speech.
- Vignette 7: When Wendy, age 7, tells a story, it's hard to follow what she's talking about. If her mother sends her upstairs for the scissors, Wendy forgets

what she wants before she gets there. However, Wendy speaks quite well with her friends and family.

Question 21. Please rate your agreement/disagreement with this statement - "There is an increased likelihood of mental health concerns in those who initially presented with significant speech/language impairments as a child".

Question 22. Please rate your agreement/disagreement with this statement - "Behavioural disorders may indicate undiagnosed communication, learning, literacy and/or attention/concentration problems".

Question 23. Please rate your agreement/disagreement with this statement - "Psychological trauma can negatively impact language development".

Question 24. Please rate your agreement/disagreement with this statement - "There are higher prevalence rates of swallowing disorders in people with diagnosed mental health conditions".

Question 25. Please rate your agreement/disagreement with this statement - "There are many DSM-5 diagnostic categories which include communication impairment in the diagnostic criteria".

Question 26. Please rate your agreement/disagreement with this statement - "Children in care are at a much greater risk of having a language impairment, social and emotional difficulties and increased risk of contact with the criminal justice system".

Question 27. Please rate your agreement/disagreement with this statement - "There is a greater need for speech pathologists to be involved in mental health care programs for children and adults".
